# Penta­carbon­yl(imidazolidine-2-thione-κ*S*)tungsten(0)

**DOI:** 10.1107/S1600536810016314

**Published:** 2010-05-08

**Authors:** Salah Merniz, Mahiedine Mokhtari, Hénia Mousser, Lahcène Ouahab, Abdelhamid Mousser

**Affiliations:** a Département de Chimie, Faculté des Sciences Exactes, Université Mentouri Constantine, Route de Ain El Bey, Constantine, Algeria; b Département de Chimie, Faculté des Sciences Exactes, Université Larbi Ben M’Hidi, Route de Constantine, Oum El Bouaghi, Algeria; c Département de Chimie Industrielle, Faculté des Sciences de l’Ingénieur, Université Mentouri Constantine, Campus Chaab Erssas, Constantine, Algeria; d Equipe Organométallique et Matériaux Moléculaires, UMR6226 CNRS-Université de Rennes 1, Avenue du Général Leclerc, 35042, Rennes, France

## Abstract

In the title complex, [W(C_3_H_6_N_2_S)(CO)_5_], the W atom displays an octa­hedral coordination with five CO mol­ecules and an imidazolidine-2-thione mol­ecule. The W(CO)_5_ unit is coordinated by the cyclic thione ligand through a W—S dative bond. The W—S and C—S bond lengths are 2.599 (2) and 1.711 (9) Å, respectively. This last distance is significantly longer than that of free cyclic thio­ureas. The geometry of the title compound suggests *sp* ^3^-hybridization of the S atom caused by the greatly polarized linkage W—S—C bond angle, which is close to tetra­hedral [109.50 (3)°]. In the crystal packing, N—H⋯O and N—H⋯S hydrogen-bonding inter­actions stabilize the structure and build up chains parallel to [101].

## Related literature

For the properties of imidazolinethio­nes or cyclic thio­ureas, see: Gok & Çetinkaya (2004 ▶); Kuhn & Kratz (1993 ▶); Reglinski *et al.* (1999 ▶); Crossley *et al.* (2006 ▶); Saito *et al.* (2007 ▶); Raper *et al.* (1983 ▶). For hydrogen-bond motifs, see: Etter *et al.* (1990 ▶); Bernstein *et al.* (1995 ▶); Beheshti *et al.* (2007 ▶). For related structures, see: Kuhn *et al.* (1998 ▶); Mak *et al.* (1985) ▶; Valdés-Martinez *et al.* (1988 ▶, 1996 ▶); Pasynsky *et al.* (2007 ▶); Darensbourg *et al.* (1999 ▶).
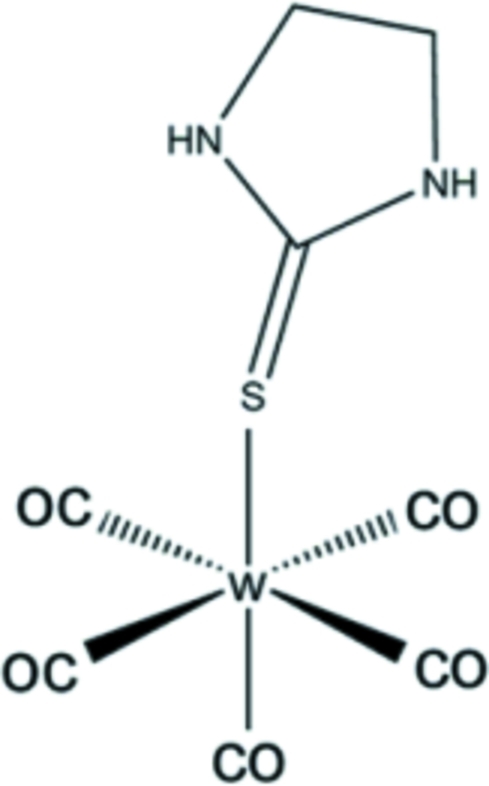


## Experimental

### 

#### Crystal data

[W(C_3_H_6_N_2_S)(CO)_5_]
*M* *_r_* = 426.06
Triclinic, 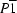

*a* = 6.652 (1) Å
*b* = 7.8120 (12) Å
*c* = 11.6240 (15) Å
α = 84.071 (5)°
β = 85.042 (6)°
γ = 87.704 (7)°
*V* = 598.27 (15) Å^3^
*Z* = 2
Mo *K*α radiation
μ = 9.84 mm^−1^
*T* = 293 K
0.08 × 0.06 × 0.04 mm

#### Data collection

Bruker SMART 1K CCD area-detector diffractometer
Absorption correction: refined from Δ*F* (cubic fit to sinθ/λ, 24 parameters; Parkin *et al.*, 1995 ▶) *T*_min_ = 0.526, *T*_max_ = 0.867
2623 measured reflections
2623 independent reflections
2324 reflections with *I* > 2σ(*I*)

#### Refinement

*R*[*F* ^2^ > 2σ(*F* ^2^)] = 0.039
*wR*(*F* ^2^) = 0.106
*S* = 1.10
2623 reflections
154 parameters
H-atom parameters constrained
Δρ_max_ = 1.44 e Å^−3^
Δρ_min_ = −1.58 e Å^−3^

### 

Data collection: *SMART* (Bruker, 2001 ▶); cell refinement: *SAINT* (Bruker, 2001 ▶); data reduction: *SAINT*; program(s) used to solve structure: *SIR92* (Altomare *et al.*, 1993 ▶); program(s) used to refine structure: *SHELXL97* (Sheldrick, 2008 ▶); molecular graphics: *ORTEPIII* (Burnett & Johnson, 1996 ▶) and *ORTEP-3 for Windows* (Farrugia, 1997 ▶); software used to prepare material for publication: *WinGX* (Farrugia, 1999 ▶), *PARST97* (Nardelli, 1995 ▶) and *Mercury* (Macrae *et al.*, 2006 ▶).

## Supplementary Material

Crystal structure: contains datablocks I, global. DOI: 10.1107/S1600536810016314/dn2555sup1.cif

Structure factors: contains datablocks I. DOI: 10.1107/S1600536810016314/dn2555Isup2.hkl

Additional supplementary materials:  crystallographic information; 3D view; checkCIF report

## Figures and Tables

**Table 1:** Hydrogen-bond geometry (Å, °)

*D*—H⋯*A*	*D*—H	H⋯*A*	*D*⋯*A*	*D*—H⋯*A*
N1—H1⋯O5^i^	0.86	2.39	3.039 (11)	133
N2—H2⋯S1^ii^	0.86	2.88	3.630 (9)	147

Symmetry codes: (i) 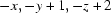; (ii) 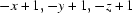.

**Fig. 1.**
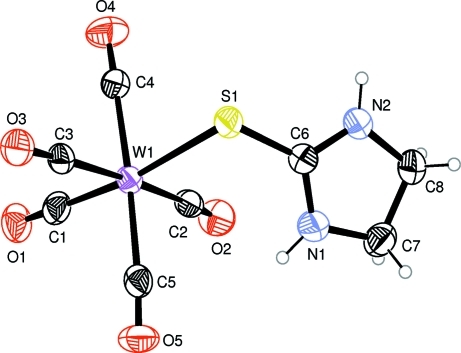
The molecular structure of (I), with atom labels and 30% probability displacement ellipsoids for non-H atoms. H atoms are represented as small spheres of arbitrary radii.

**Fig. 2.**
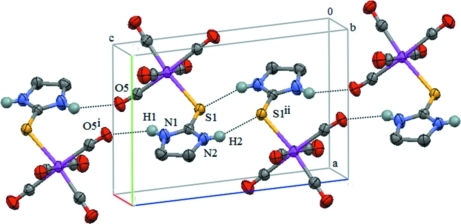
Partial packing view of (I) showing the chain formed by N-H···O and N-H···S hydrogen bonds shown as dashed lines. H atoms not involved in hydrogen bonding have been omitted for clarity. [Symmetry codes: (i) -x, -y+1, -z+2; (ii) -x+1, -y+1, -z+1].

## supplementary crystallographic information

### Comment

Imidazolinethiones or cyclic thioureas are an important classe of compounds with a wide variety of applications (Gok & çetinkaya, 2004; Kuhn & Kratz, 1993). The chemical interests of these cyclic thioureas lie in their face capping character, in their structural analogy with thiolated nucleosides and in their application to enzyme models (Reglinski *et al.*, 1999; Crossley *et al.*, 2006; Saito *et al.*, 2007). The diverse properties of the cyclic thioureas have been attributed to the coordination ability of the heterocyclic RN—C(S)—NR' thioamide group, as a monodentate ligand, to both metallic and non-metallic elements, leading to stable electron donor–acceptor complexes (Raper *et al.*, 1983). Our research has been focused for some time on coordination compounds of sulfur containing ligands with carbonyl metals. The structure of the Imidazolidine-2-Thione-W(CO)_5_ complex (I), was carried out and results are presented here.

The tungsten atom displays octahedral geometry with five CO and the Imidazolidine-2-Thione molecules (Fig. 1). The bond distances and angles in (I) are within normal range and are comparable to the corresponding values observed in similar structures (Saito *et al.*, 2007; Mak *et al.*, 1985; Valdés-Martinez *et al.*, 1988; Valdés-Martinez *et al.*, 1996; Pasynsky *et al.*, 2007; Darensbourg *et al.*, 1999). Such geometry of (I) suggests sp3 hybridization of the sulfur atom caused by the greatly polarized M—S—C linkage. Indeed, the W—S—C bond angles is 109.50 (3)° and is close to a tetrahedral angle. As expected, the C=S bond is elongated and the C(6)—S(1) interatomic distance is 1.711 (9) Å and it is significantly longer than that of free cyclic thiourea, 1.690 (2) Å (Mak *et al.*, 1985; Kuhn *et al.*, 1998). The bond length between the metal and trans-carbonyl carbon atoms is 1.970 (10) Å. This is substantially shorter than the metal cis carbonyl bonds. The average of the separations between the metal and cis carbonyls is 2.049 Å.

Intermolecular N—H···O hydrogen bonds generate R_2_^2^(14) graph-set motif (Etter *et al.*, 1990; Bernstein *et al.*, 1995) resulting in the formation of a pseudo dimer. Further N-H···O [R_2_^2^(14)] and N—H···S [R_2_^2^(8)] interactions link these dimers forming chains parallel to the [1 0 1] direction (Table 1, Fig.2). The N-H···S hydrogen bond distance is in the same range of there observed in the heterocyclic thione complexes (Beheshti *et al.*, 2007).

### Experimental

A solution of W(CO)_6_ (527 mg, 1.5 mmol) and Imidazolidine-2-thione (153 mg, 1.5 mmol) in 40 ml of dry THF was irradiated for 2 h with vigorous stirring. The excess of W(CO)_6_ was mouved by filtration and the solvent was evaporated under reduced pressure. The residue was recrystallised from THF/hexane (1:5 ratio). Bright yellow crystals were washed three times with portions of hexane, and dried under vacuum. Yield:(34%).

### Refinement

H atoms were positined geometrically, using a riding model with C—H = 0.96 Å (*U*_iso_(H) = 1.5) (including free rotation about C—C and C—N bond) for methyl groups and with C—H = 0.93 and 0.97 Å (1.2 for aromatic and methylene groups) times *U*_eq_(C).

### Crystal data

**Table:**

[W(C_3_H_6_N_2_S)(CO)_5_]	*Z* = 2
*M**_r_* = 426.06	*F*(000) = 396
Triclinic, *P*1	*D*_x_ = 2.365 Mg m^−^^3^
Hall symbol: -P 1	Mo *K*α radiation, λ = 0.71073 Å
*a* = 6.652 (1) Å	Cell parameters from 9229 reflections
*b* = 7.8120 (12) Å	θ = 1.0–27.1°
*c* = 11.6240 (15) Å	µ = 9.84 mm^−^^1^
α = 84.071 (5)°	*T* = 293 K
β = 85.042 (6)°	Prism, yellow
γ = 87.704 (7)°	0.08 × 0.06 × 0.04 mm
*V* = 598.27 (15) Å^3^	

### Data collection

**Table:**

Bruker SMART 1K CCD area-detector diffractometer	2623 independent reflections
Radiation source: fine-focus sealed tube	2324 reflections with *I* > 2σ(*I*)
graphite	*R*_int_ = 0.0000
Detector resolution: 8.192 pixels mm^-1^	θ_max_ = 27.2°, θ_min_ = 3.0°
ω scan	*h* = −8→8
Absorption correction: part of the refinement model (Δ*F*) (cubic fit to sinθ/λ, 24 parameters; Parkin *et al.*, 1995)	*k* = −9→10
*T*_min_ = 0.526, *T*_max_ = 0.867	*l* = 0→14
2623 measured reflections	

### Refinement

**Table:**

Refinement on *F*^2^	Primary atom site location: structure-invariant direct methods
Least-squares matrix: full	Secondary atom site location: difference Fourier map
*R*[*F*^2^ > 2σ(*F*^2^)] = 0.039	Hydrogen site location: inferred from neighbouring sites
*wR*(*F*^2^) = 0.106	H-atom parameters constrained
*S* = 1.10	*w* = 1/[σ^2^(*F*_o_^2^) + (0.0555*P*)^2^ + 0.8559*P*] where *P* = (*F*_o_^2^ + 2*F*_c_^2^)/3
2623 reflections	(Δ/σ)_max_ = 0.001
154 parameters	Δρ_max_ = 1.44 e Å^−^^3^
0 restraints	Δρ_min_ = −1.58 e Å^−^^3^

### Special details

**Table:**

Geometry. All esds (except the esd in the dihedral angle between two l.s. planes) are estimated using the full covariance matrix. The cell esds are taken into account individually in the estimation of esds in distances, angles and torsion angles; correlations between esds in cell parameters are only used when they are defined by crystal symmetry. An approximate (isotropic) treatment of cell esds is used for estimating esds involving l.s. planes.
Refinement. Refinement of *F*^2^ against ALL reflections. The weighted *R*-factor wR and goodness of fit *S* are based on *F*^2^, conventional *R*-factors *R* are based on *F*, with *F* set to zero for negative *F*^2^. The threshold expression of *F*^2^ > σ(*F*^2^) is used only for calculating *R*-factors(gt) etc. and is not relevant to the choice of reflections for refinement. *R*-factors based on *F*^2^ are statistically about twice as large as those based on *F*, and *R*- factors based on ALL data will be even larger.

### Fractional atomic coordinates and isotropic or equivalent isotropic displacement parameters (Å^2^)

**Table:**

	*x*	*y*	*z*	*U*_iso_*/*U*_eq_	
W1	0.04631 (4)	0.22504 (4)	0.75225 (2)	0.05469 (14)	
S1	0.1747 (3)	0.4882 (3)	0.61423 (18)	0.0631 (5)	
O1	−0.0857 (14)	−0.1215 (10)	0.8903 (7)	0.093 (2)	
O2	0.4366 (12)	0.1909 (13)	0.8942 (7)	0.097 (2)	
O3	−0.3638 (10)	0.2376 (10)	0.6320 (6)	0.0757 (17)	
O4	0.2472 (17)	0.0053 (12)	0.5555 (8)	0.106 (3)	
O5	−0.1845 (13)	0.4202 (12)	0.9529 (6)	0.088 (2)	
N1	0.3318 (15)	0.6250 (13)	0.7912 (7)	0.088 (3)	
H1	0.2360	0.5882	0.8409	0.106*	
N2	0.5082 (13)	0.6600 (11)	0.6281 (7)	0.075 (2)	
H2	0.5434	0.6543	0.5556	0.090*	
C1	−0.0386 (15)	0.0069 (13)	0.8400 (8)	0.071 (2)	
C2	0.3013 (13)	0.2079 (12)	0.8406 (7)	0.0634 (19)	
C3	−0.2170 (11)	0.2399 (10)	0.6719 (7)	0.0533 (15)	
C4	0.1775 (14)	0.0833 (12)	0.6262 (8)	0.0626 (19)	
C5	−0.0988 (13)	0.3583 (12)	0.8803 (8)	0.0624 (19)	
C6	0.3454 (12)	0.5942 (10)	0.6806 (7)	0.0587 (17)	
C7	0.4921 (17)	0.7255 (14)	0.8196 (9)	0.077 (2)	
H3	0.5645	0.6660	0.8816	0.093*	
H4	0.4420	0.8365	0.8422	0.093*	
C8	0.6264 (17)	0.7454 (15)	0.7050 (9)	0.079 (3)	
H5	0.6447	0.8656	0.6773	0.094*	
H6	0.7574	0.6884	0.7130	0.094*	

### Atomic displacement parameters (Å^2^)

**Table:**

	*U*^11^	*U*^22^	*U*^33^	*U*^12^	*U*^13^	*U*^23^
W1	0.0489 (2)	0.0648 (2)	0.0505 (2)	−0.00673 (13)	−0.00158 (12)	−0.00630 (13)
S1	0.0636 (11)	0.0727 (12)	0.0541 (10)	−0.0159 (9)	−0.0067 (8)	−0.0045 (9)
O1	0.103 (6)	0.088 (5)	0.085 (5)	−0.032 (4)	−0.016 (4)	0.016 (4)
O2	0.069 (4)	0.141 (7)	0.085 (5)	0.002 (4)	−0.029 (4)	−0.010 (5)
O3	0.057 (3)	0.099 (5)	0.074 (4)	−0.006 (3)	−0.011 (3)	−0.012 (3)
O4	0.126 (7)	0.106 (6)	0.086 (5)	0.018 (5)	0.010 (5)	−0.040 (5)
O5	0.089 (5)	0.109 (5)	0.066 (4)	0.010 (4)	0.010 (4)	−0.033 (4)
N1	0.088 (6)	0.119 (7)	0.063 (4)	−0.038 (5)	0.005 (4)	−0.024 (4)
N2	0.078 (5)	0.090 (5)	0.059 (4)	−0.030 (4)	0.002 (4)	−0.009 (4)
C1	0.070 (5)	0.080 (6)	0.068 (5)	−0.013 (4)	−0.020 (4)	−0.010 (4)
C2	0.057 (4)	0.079 (5)	0.054 (4)	−0.006 (4)	0.001 (3)	−0.006 (4)
C3	0.042 (3)	0.064 (4)	0.055 (4)	−0.006 (3)	−0.004 (3)	−0.007 (3)
C4	0.059 (4)	0.070 (5)	0.060 (4)	−0.006 (4)	−0.004 (4)	−0.009 (4)
C5	0.054 (4)	0.077 (5)	0.057 (4)	−0.012 (4)	−0.012 (3)	−0.001 (4)
C6	0.061 (4)	0.059 (4)	0.056 (4)	−0.004 (3)	−0.002 (3)	−0.006 (3)
C7	0.080 (6)	0.079 (6)	0.078 (6)	−0.008 (5)	−0.012 (5)	−0.022 (5)
C8	0.077 (6)	0.085 (6)	0.077 (6)	−0.031 (5)	−0.003 (5)	−0.012 (5)

### Geometric parameters (Å, °)

**Table:**

W1—C1	1.970 (10)	N1—C6	1.327 (11)
W1—C4	2.042 (9)	N1—C7	1.430 (13)
W1—C3	2.049 (7)	N1—H1	0.8598
W1—C2	2.050 (9)	N2—C6	1.294 (11)
W1—C5	2.055 (10)	N2—C8	1.465 (12)
W1—S1	2.599 (2)	N2—H2	0.8601
S1—C6	1.711 (9)	C7—C8	1.536 (17)
O1—C1	1.148 (11)	C7—H3	0.9700
O2—C2	1.134 (12)	C7—H4	0.9700
O3—C3	1.118 (10)	C8—H5	0.9700
O4—C4	1.130 (12)	C8—H6	0.9700
O5—C5	1.118 (12)		
			
C1—W1—C4	87.8 (4)	C8—N2—H2	123.5
C1—W1—C3	89.7 (3)	O1—C1—W1	178.9 (10)
C4—W1—C3	89.5 (3)	O2—C2—W1	175.7 (9)
C1—W1—C2	88.5 (4)	O3—C3—W1	175.3 (8)
C4—W1—C2	92.6 (4)	O4—C4—W1	178.8 (9)
C3—W1—C2	177.1 (3)	O5—C5—W1	175.0 (9)
C1—W1—C5	89.7 (4)	N2—C6—N1	109.4 (8)
C4—W1—C5	176.8 (3)	N2—C6—S1	124.2 (7)
C3—W1—C5	88.5 (3)	N1—C6—S1	126.3 (7)
C2—W1—C5	89.3 (3)	N1—C7—C8	102.3 (8)
C1—W1—S1	172.4 (3)	N1—C7—H3	111.3
C4—W1—S1	84.6 (3)	C8—C7—H3	111.3
C3—W1—S1	89.4 (2)	N1—C7—H4	111.3
C2—W1—S1	92.8 (3)	C8—C7—H4	111.3
C5—W1—S1	97.8 (3)	H3—C7—H4	109.2
C6—S1—W1	109.5 (3)	N2—C8—C7	101.7 (8)
C6—N1—C7	113.4 (8)	N2—C8—H5	111.4
C6—N1—H1	123.3	C7—C8—H5	111.4
C7—N1—H1	123.3	N2—C8—H6	111.4
C6—N2—C8	113.0 (8)	C7—C8—H6	111.4
C6—N2—H2	123.5	H5—C8—H6	109.3

### Hydrogen-bond geometry (Å, °)

**Table:**

*D*—H···*A*	*D*—H	H···*A*	*D*···*A*	*D*—H···*A*
N1—H1···O5^i^	0.86	2.39	3.039 (11)	133
N2—H2···S1^ii^	0.86	2.88	3.630 (9)	147

Symmetry codes: (i) −*x*, −*y*+1, −*z*+2; (ii) −*x*+1, −*y*+1, −*z*+1.
